# Small RNA Sequencing Reveals Regulatory Roles of MicroRNAs in the Development of *Meloidogyne incognita*

**DOI:** 10.3390/ijms20215466

**Published:** 2019-11-02

**Authors:** Huawei Liu, Robert L. Nichols, Li Qiu, Runrun Sun, Baohong Zhang, Xiaoping Pan

**Affiliations:** 1 College of Life Sciences, Northwest A&F University, Yangling 712100, China; bioche@nwsuaf.edu.cn; 2 Department of Biology, East Carolina University, Greenville, NC 27858, USA; QL525@126.com (L.Q.); sunrunrun123@163.com (R.S.); zhangb@ecu.edu (B.Z.); 3 Cotton Incorporated, Cary, NC 27513, USA; 4 College of Veterinary Medicine, Northwest A&F University, Yangling 712100, China; 5 Henan Institute of Science and Technology, Xinxiang 453003, China

**Keywords:** root-knot nematode, cotton infection, small RNA, gene regulation, deep sequencing

## Abstract

MicroRNAs (miRNAs) are an extensive class of small regulatory RNAs. Knowing the specific expression and functions of miRNAs during root-knot nematode (RKN) (*Meloidogyne incognita*) development could provide fundamental information about RKN development as well as a means to design new strategies to control RKN infection, a major problem of many important crops. Employing high throughput deep sequencing, we identified a total of 45 conserved and novel miRNAs from two developmental stages of RKN, eggs and J2 juveniles, during their infection of cotton (*Gossypium hirsutum L.*). Twenty-one of the miRNAs were differentially expressed between the two stages. Compared with their expression in eggs, two miRNAs were upregulated (miR252 and miRN19), whereas 19 miRNAs were downregulated in J2 juveniles. Nine miRNAs were expressed at high levels, with >1000 reads per mapped million (RPM) sequenced reads in both eggs and J2 juveniles (miR1, miR124, miR2-3p, miR252, miR279, miR57-5p, miR7904, miR87, and miR92). Three miRNAs were only expressed in eggs (miR4738, miRN3, and miRN5). These differentially expressed miRNAs may control RKN development by regulating specific protein-coding genes in pathways associated with RKN growth and development.

## 1. Introduction

Parasitic nematodes are pests affecting crop yields and quality in many important crops such as cotton (*Gossypium hirsutum* L.), the most economically significant fiber crop. The most damaging nematode parasites in the U.S. cotton crop are root-knot (*Meloidogyne incognita*), reniform (*Rotylenchulus reniformis*), and sting (*Belonolaimus longicaudatus*) nematodes [1]. The southern root-knot nematode (RKN) *M. incognita* is considered the most damaging in part because it occurs widely from California to North Carolina. Infection of *M. incognita* to plant roots is intimately involved in its life-cycle. The infective second-stage juvenile (J2) enters plant roots and migrates to the vascular tissues, where it transforms certain vascular cells into multinucleate and hypertrophied so-termed “giant cells”, which become the nematodes’ feeding and reproduction sites. To transform and maintain the giant cells, the nematodes secrete effector proteins into the plants via the stylet in their mouth parts [2,3,4]. The nematode will then become sedentary, use the giant cells as feeding sites, go through J3 and J4 molts, and develop into a reproductive female which will lay thousands of eggs. The infected roots will become gnarled; the feeding sites are colloquially called “knots” [5]. Some genes coding for the effector proteins that facilitate parasitism have been identified [6]; however, the regulation of such gene expression remains unclear. 

MicroRNAs (miRNAs) are an extensive class of small (~21 nt) noncoding endogenous RNA molecules. miRNA-mediated gene silencing is a fundamental regulatory mechanism of gene expression. miRNAs inhibit gene expression by binding to specific sites located at the 3′ untranslated region (UTR) of the target mRNAs and degrade or inhibit its translation. More than 30% of protein-coding genes may be regulated by miRNAs [7,8]. Thus, miRNAs play a significant role in the regulation of almost all critical biological processes in plant and animals; they act as regulatory switches, controlling reproduction and developmental timing, signal transduction, cell fate, apoptosis, and response to environmental stressors [9,10,11]. Knowledge of individual miRNA regulation in nematodes has been primarily obtained from model, free-living species such as *Caenorhabditis elegans* and some animal parasites. For example, the miRNAs *lin-4* and *let-7* function as key regulators of developmental timing in *C. elegans*. *Lin 4* controls early stage development in *C. elegans* [12,13], while miRNA *let-7* mediates transition of *C. elegans* larvae to adults [9,14]. Aberrant expression of either of these miRNAs causes aberrant development and growth of *C. elegans*. miRNAs function by targeting protein-coding genes. For example, miRNA *lin-4* targets *lin-14*, which regulates the timing of cell division during postembryonic development [12]. The miRNA *let-7* targets *hbl-1*, which regulates developmental timing and affects locomotion and egg-laying [9,14]. However, much work on the role of miRNAs in the major agricultural pest nematode, *M. incognita* has not been accomplished. With the draft genome of *M. incognita* available [5], it may be possible to identify miRNAs in *M. incognita* and further study their functions in regulating reproduction, development, metabolism, and parasitism. Our previous work and that of others have identified *M. incognita* miRNAs isolated from pepper roots (*Capsicum annuum* L.) and identified certain miRNAs that were conserved in other parasitic nematodes [15,16]. It is known that expression of miRNAs corresponds with developmental stages and can be induced by biotic and abiotic stresses. To date, there has been no study of the miRNA expression profile of *M. incognita* concurrent with its infection of cotton. Additionally, there is no study reporting the temporal miRNA expression changes at different developmental stages of *M. incognita*. 

Several reports indicate the benefits and efficiency of using RNAi-related strategies for controlling critical gene functions in nematodes [17,18,19,20], highlighting the importance of better understanding the process of small RNA regulation of gene expression in RKN. Our results have potential to be used in integrated pest management programs and together with biopesticides [21] and basic substances [22].

## 2. Results 

### 2.1. Small RNA Deep Sequencing of Eggs and J2 Juveniles

Deep sequencing provides a powerful tool to sequence and identify all potential small RNAs, including microRNAs (miRNAs). All small RNAs from *M. incognita* obtained at the two developmental stages (eggs and J2) were sequenced. A total of 19,952,271 and 17,526,380 reads were obtained for the egg and J2 juvenile stages, respectively. A majority of reads (>98%) were clean with high quality; a total of 33.83 million clean reads were obtained, for each sample no less than 15 million clean reads were retrieved, as seen in Table 1. The size distribution of small RNAs are similar at both stages assessed.

**Figure 1:**
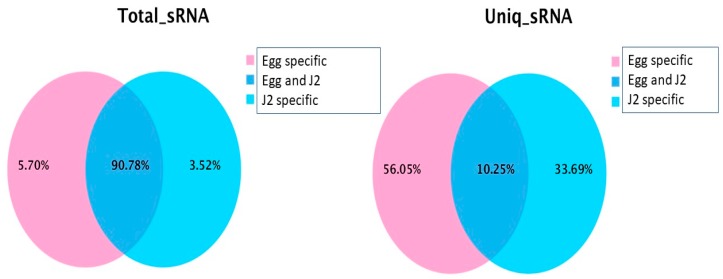
Sequence comparison between eggs (Pink) and J2 larvae (Blue). (**Left**): total clean reads; (**right**): the unique small RNA sequences.

Among the 33,827,418 total clean reads from all samples, more than 90% (30,709,303) were common between the egg and J2 stages, as seen in Figure 1. However, only 10% of unique sequences were common between the two stages, as seen in Figure 1. Thus, the majority of expressed small RNAs are common among the two RKN developmental stages since only 5.7% of the sequence were expressed in eggs but not in J2, and only 3.5% of the expressed sequences were in J2 but not in eggs. However, the types of expressed small RNAs (unique sequences) were significantly different between the two RKN developmental stages. Only 10.3% of small RNAs were expressed commonly in both eggs and J2s. More than half (56.1%) of the small RNAs were only expressed in eggs but not in J2s, whereas 33.7% of the small RNAs were only expressed in J2s but not in eggs.

**Figure 2:**
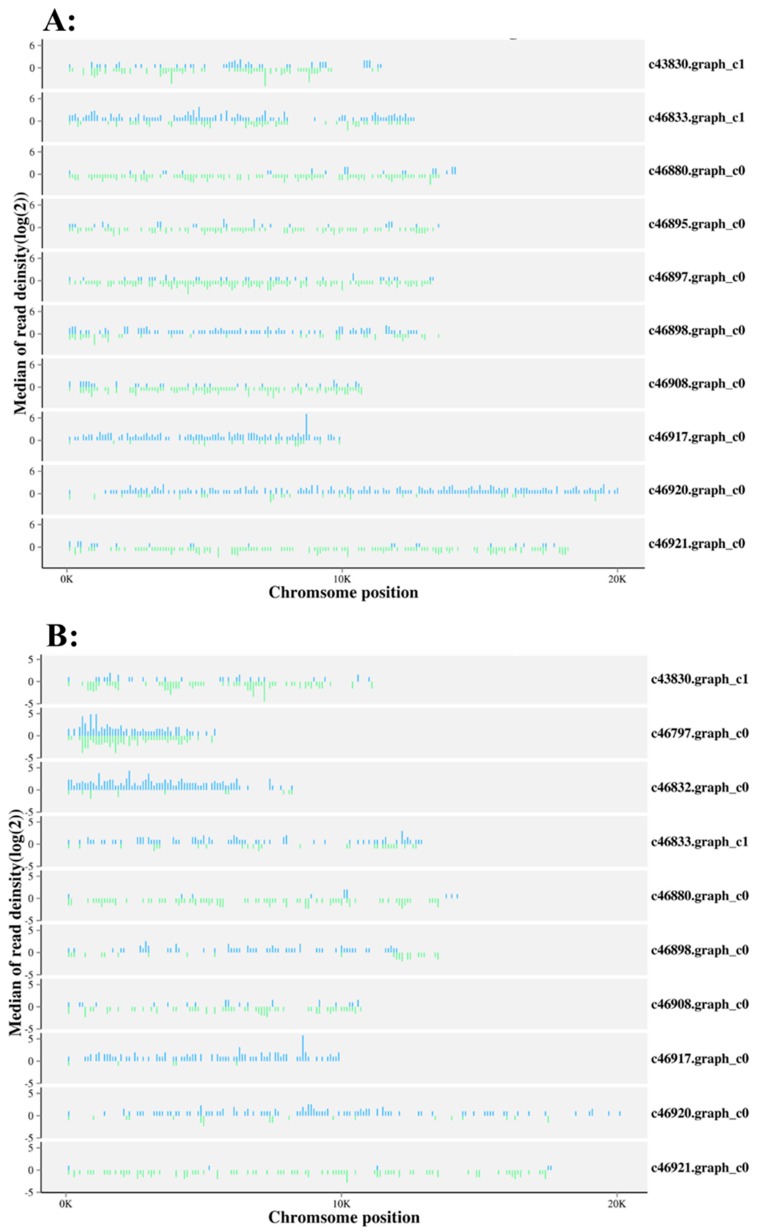
Genome-wide distribution of read coverage on each chromosome. X-axis: location of reads at each chromosome; Y-axis, the coverage intensity in Log2 value. (**A**) Eggs (**B**) J2 Juveniles.

The locations of sequenced small RNAs also provide evidence for small RNA expression. Figure 2 shows the chromosome distribution and expression of small RNA population is different between *M. incognita* eggs and J2 juveniles. miRNAs are spread throughout the *M. incognita* genome. 

### 2.2. Characterization of Small RNAs in RKN Eggs and J2 Larvae

rRNAs account for about 20% of the identified sequenced small RNAs. About 1% of the sequenced small RNAs were tRNAs; these tRNAs occurred in both eggs and J2s, as seen in Table 2. About 0.01% of small RNAs were snoRNAs. More than 80% of the putative small RNAs were not aligned to any currently-known small RNAs. 

### 2.3. Identification and Specific Expression of M. Incognita miRNAs at Eggs and J2 Stages

High throughput deep sequencing technology identified 24 conserved and 21 novel *M. incognita* miRNAs that were expressed following cotton infection. Forty-five identified miRNAs were identified from eggs and 42 of the same miRNAs were also found in J2s. Three miRNAs identified from eggs were not found in J2 juveniles: miR4738, miR N3, and miR N5.

**Figure 3:**
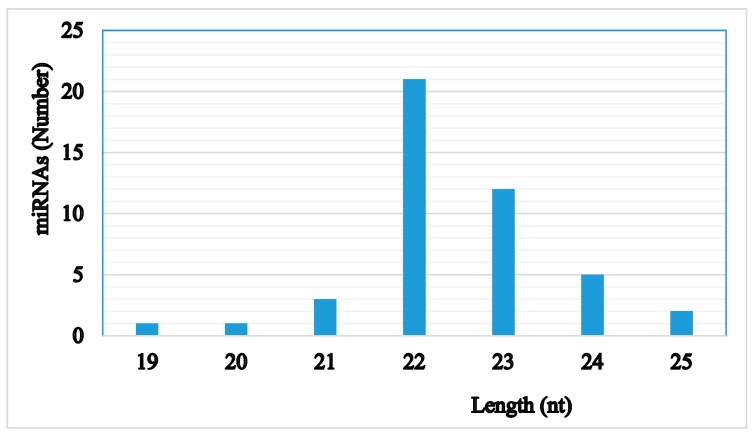
Size distribution (nucleotide length) of identified miRNAs from *M. incognita*.

**Figure 4:**
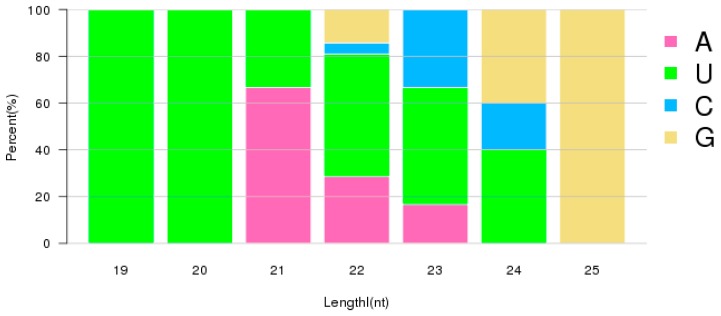
The percentage of the first nucleotide at the 5′ end of identified RKN miRNAs.

**Figure 5:**
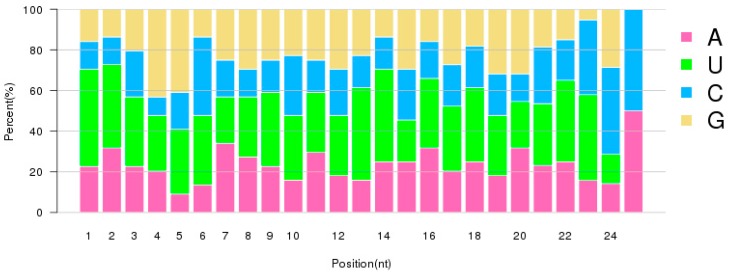
The nucleotide percentage at each position of identified RKN miRNAs.

The majority of identified miRNAs were of 22 nt in length. The next largest size category was those of 23 nt length, as seen in Figure 3. This size distribution is similar to that reported in other organisms. The first nucleotide at the 5′ end of a mature miRNA is dominated by nucleotide U, particularly for miRNAs with lengths of 19–24, as seen in Figure 4. Although nucleotides also show bias among positions, the differences are not as significant as they were in the first position, as seen in Figure 5. During miRNA biogenesis and function, there is a nucleotide bias among different positions, which serves as the signal for Dicer cutting and recognizing target mRNA sequences.

**Figure 6:**
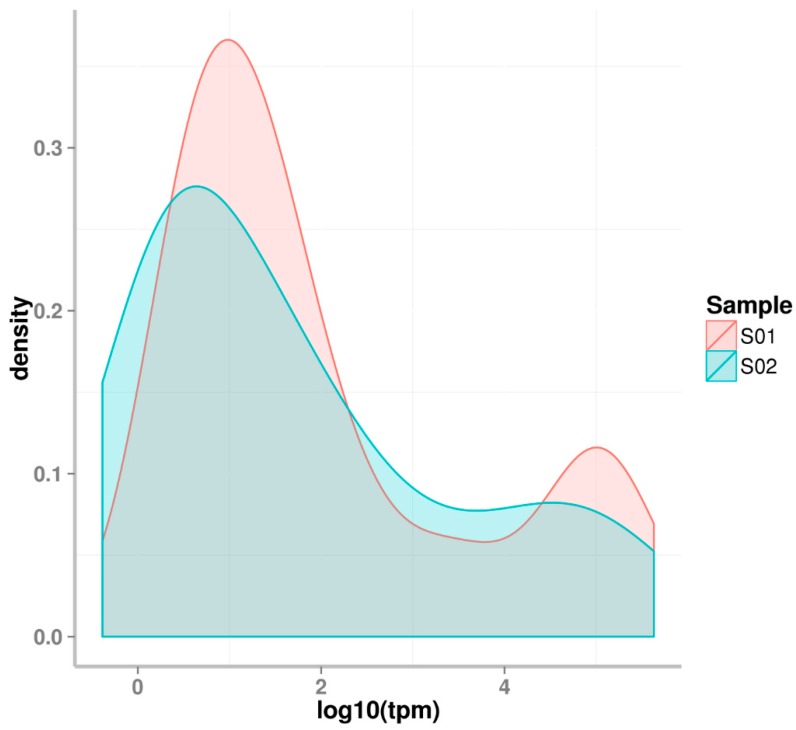
The expression pattern of miRNAs in two developmental stages of RKN. S01: eggs; S02: J2 juveniles. The X-axis means the log10 of the relative expression that was presented by the reads per million mapped total reads.

The expression patterns differed between the two developmental stages of *M. incognita*, as seen in Figure 6. Although the majority of miRNAs had low expression levels, the number of miRNAs with low expression levels was greater in eggs than that in J2 Juveniles. During the egg stage, there were a lesser number of miRNAs with medium expression levels than that there were during the J2 stage. About 10% of miRNAs had high expression levels in the egg stage. Only 5–7% of miRNAs were highly expressed in the J2 stage. In RKN eggs, miRNAs were generally divided into two groups, one group with high expression levels, and the second with relatively low expression levels. In the RKN J2 stage, the majority of miRNAs were expressed at low levels.

Following cotton infection, expression levels of individual miRNAs differed significantly among the 45 identified miRNAs, as seen in Table 3. In eggs, 30 of 45 (~67%) identified miRNAs were sequenced with less than 100 total reads per million (TPM). In contrast, 11 miRNAs were sequenced with more than 1000 TPM: miR1, miR124, miR239b, miR2-3p, miR252, miR279, miR57-5p, miR7904, miR87, miR92, and miR993-3p. In the J2 stage, 27 of 42 (~64%) identified miRNAs were sequenced with less than 100 TPM; while as in eggs approximately one third or nine miRNAs were expressed at higher than 1000 TPM: miR1, miR124, miR2-3p, miR252, miR279, miR57-5p, miR7904, miR87, and miR92. In eggs, the top three most commonly expressed miRNAs were miR1, miR92, and miR279 with 244,811, 221,662, and 188,859 TPM, respectively. The top three most highly expressed miRNAs in J2 juveniles were miR1, miR92, and miR124 with 426,558, 317,693, and 88,381 TPM, respectively. This analysis shows that following infection, miR1 and miR92 are the most abundant miRNAs at both *M. incognita* developmental stages. 

**Figure 7:**
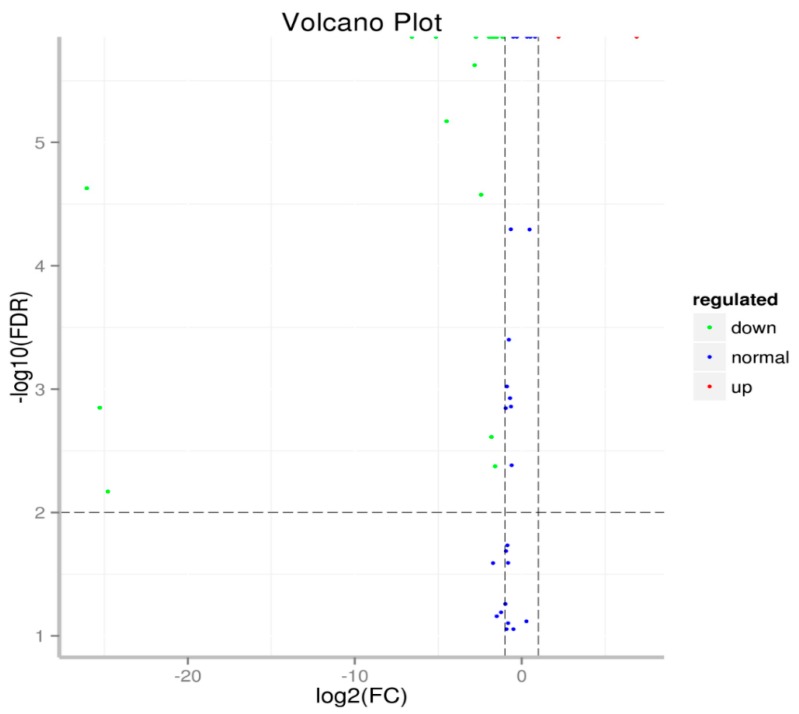
Volcano plot of microRNA expression in two developmental stages of RKN. In this figure, each spot represents an individual miRNA. The x-axis represents the fold change of each miRNA at log 2 level between eggs and J2 juveniles. A bigger number means a bigger fold change. The y-axis represents the significance level, a bigger number means higher significance. An miRNA in blue means that it has no significant change between the two developmental stages; the red spots mean significantly upregulated miRNAs in J2 juveniles compared with in eggs (*p* < 0.01); the green spots mean significantly downregulated miRNA in the J2 stage (*p* < 0.01).

Of the 45 identified, conserved, and novel miRNAs in RKN, 21 were expressed differently between the two developmental stages (*p* < 0.01, Table 4 and Figure 7). Compared with the expression level of miRNAs in eggs, two miRNAs were significantly upregulated—miR252 and miRN19. miR252 was among the most highly expressed miRNAs in both eggs and J2, with 1541 and 7126 TPM, respectively. Nineteen miRNAs were downregulated in the J2 stage compared to their respective levels in eggs, as seen in Table 4. Among these, miR2-3p, miR239b, miR279, miR57-5p, miR7904, and miR993-3p are highly expressed miRNAs in eggs with TPM > 1000. The miRNAs miR2-3p and miR279 were highly expressed, with more than 25,000 TPM in both eggs and the J2 stage. These same miRNAs were downregulated by 3.3- and 3.9-fold in the J2 stage as compared to their levels in the egg stage. The TPMs for miR239b dropped from 70,511 in eggs to 737 in J2, and for miR993-30 dropped from 2726 in eggs to 796 in J2. Although the expression of miR7904 was only two-fold different between the egg and the J2 larval stage, the miRNA was highly expressed in both developmental stages with more than 10,000 TPM.

Three miRNAs were only expressed in eggs (miR4738, miRN3, and miRN5) but not in the J2 larval stage; all miRNAs identified in the J2 stage also were expressed in eggs. 

### 2.4. Identification and Functional Analysis of miRNA Targets

miRNAs function through targeting protein-coding genes. Thus, identification of miRNA targets is essential for understanding miRNA functions. Through the commonly used computational programs, miRanda and RNAhybrid, a total of 547 miRNA gene targets were identified for the identified *M. incognita* miRNAs. Among the 45 identified miRNAs, 36 miRNAs have been identified to target at least one protein-coding gene. After alignment against different databases, 344 miRNA targets were annotated, as seen in Table 5.

**Figure 8:**
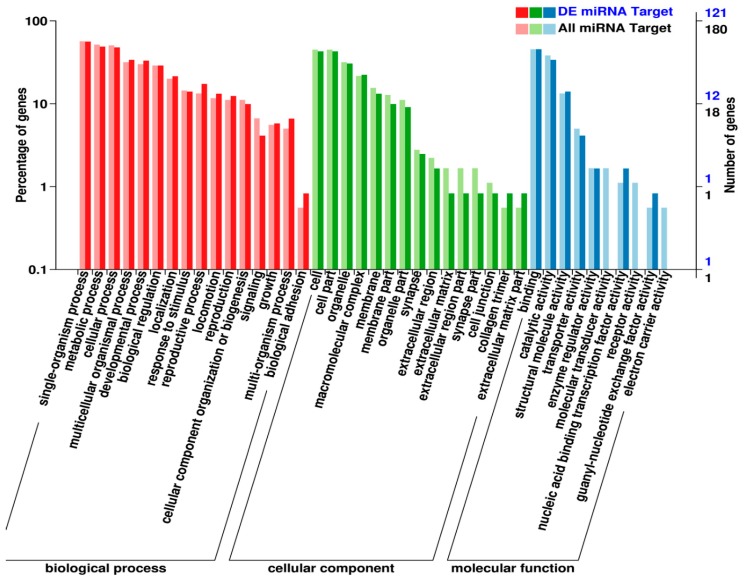
Gene ontology (GO) analysis of miRNA targets in *M. incognita*. DE miRNA target: Protein coding genes targeted by differentially expressed miRNAs. All miRNA target: Protein-coding genes targeted by all expressed miRNAs.

RKN miRNA targets are involved with biological processes, cellular components, and molecular functions, as seen in Figure 8. For the biological processes, miRNAs mostly target single-organism processes, followed by metabolic processes and cellular processes; miRNAs also target developmental processes and biological regulation, as well as response to stimulus and reproductive processes. For the cellular component, miRNAs target cell parts and organelles; miRNAs also target cell junctions and collagen trimers. For the molecular functions, the top 10 miRNA targets are binding, catalytic activity, structure molecule activity, transporter activity, enzyme regulator activity, molecular transducer activity, nucleic acid binding transcription factor activity, receptor activity, guanyl-nucleotide exchange factor activity, and electron carrier activity.

**Figure 9:**
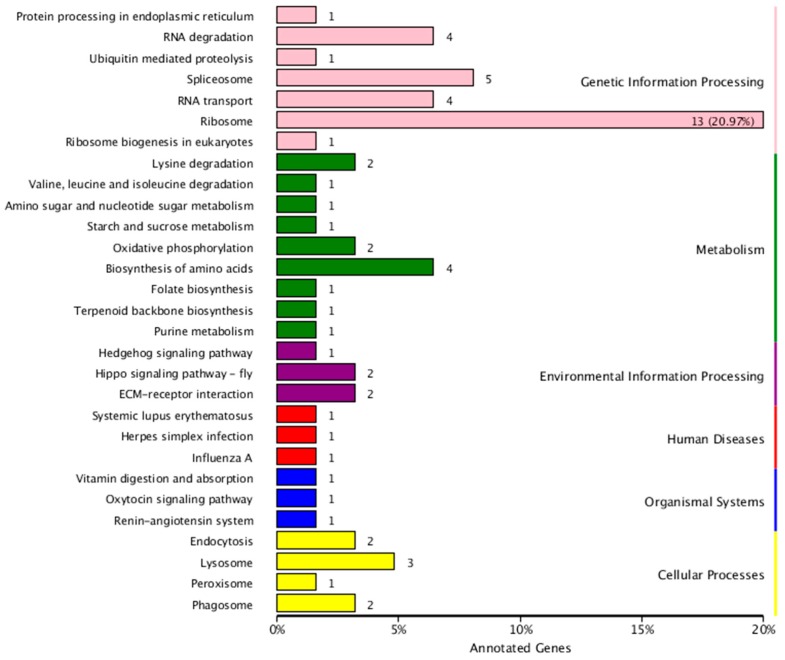
KEGG analysis of miRNA targets in RKN.

Based on Kyoto Encyclopedia of Genes and Genomes (KEGG) analysis, the miRNA targets include genetic information processing, metabolism, environmental information processing, disease, organismal systems, and cellular processes, as seen in Figure 9. miRNA-involved genetic information processing includes RNA transport and degradation, and ribosome and spliceosome activity. The miRNA targets also include many metabolic pathways, including carbohydrate, amino acid, and nucleotide metabolism. 

## 3. Discussion

Deep sequencing is a valuable tool to determine the temporal expression of individual miRNAs at different developmental stages or under specific treatments/environments. In this study, the sequence read number was used to quantitatively compare relative miRNA expression levels in different developmental stages of the parasite *M. incognita*, which infects cotton roots. Based on the total ~33.83 million clean reads obtained from deep sequencing, we identified 45 miRNAs expressed in the egg and at J2 developmental stages that comprised 24 conserved and 21 novel miRNAs. Twenty-one miRNAs were expressed differently between the two different developmental stages, eggs and J2 juveniles. Two miRNAs were upregulated and 19 were downregulated in the J2 compared to the egg stage. This, in general, suggested less miRNA inhibition of protein-coding genes in J2 as compared to eggs; therefore, many genes may be upregulated to adapt to the new parasitic developmental stage of the J2. In a previous report, Subramanian and colleagues (2016) also employed deep sequencing technology to identify miRNAs from different developmental stage of *M. incognita* nematodes collected from tomato roots [23]. They found many common miRNAs in different developmental stages as well as several development-dependent miRNAs. Our results are similar as Subramanian’s results. This suggests the miRNA-mediated mechanism is the same, regardless of the plant species infected by *M. incognita*.

The two miRNAs most commonly expressed highly in eggs and J2s were miR-1 and miR-92, with 244,811 and 221,662 TPM in eggs, respectively. These miRNAs were then more expressed at 426,558 and 317,693 (TPM) in the J2 stage, respectively. This finding indicates that these two miRNAs are fundamental to *M. incognita* development during cotton infection. miR1 is a muscle-specific miRNA that is highly conserved across worms (helminths) and vertebrates (cordates). This miRNA controls formation of the nicotinic acetylcholine receptor (nAChR) subunits at the neuromuscular junction, thus affecting cholinergic neurotransmission and muscle development [24]. In infectious rat lung work involving the nematode *Angiostrongylus cantonensis*, a pathogenic species causing eosinophilic meningitis in humans, the miR-1 expression level significantly increases during the infectious stage from larvae to young adults, and is the highest expressed miRNA in *A. cantonensis* during its parasitic cycle. Furthermore, the miR-1 is dysregulated in various cancers as a tumor-suppressive miRNA that targets multiple genes [25,26]. Overexpression of miR-1 is associated with increased chemosensitivity to anticancer drugs [27], and the downregulation of miR-1 is associated with the exposure to carcinogenic agents [28]. In addition, the miRNAs miR-1, miR-124, and miR2-3p, all of which were highly expressed in the egg and J2 juvenile stages, are conserved in both parasitic nematodes, *Ascaris suum* and *Brugia malayi*, and the free-living *C. elegans*.

The second most expressed miRNA, miR-92, is less studied in nematodes. miR-92 is an important regulator in the early development of zebrafish (*Danio rerio*) development, mediating endoderm formation and left-right asymmetry [29]. In the early development of *Drosophila melanogaster*, miR-92 is critical for neuroblast self-renewal in the larval brain by inhibiting premature differentiation [30]. miR-92 also has a role in tumorigenesis as an oncogenic regulator, targeting antiapoptotic BCL-2 [31,32]. Interestingly, despite being highly expressed in *M. incognita*, miR-92 is absent in the genome of free-living nematodes such as *C. elegans* [16], suggesting that it may be associated with parasitism. 

The conserved, immunity-related miR-279 was highly expressed in eggs with 188,859 TPM in eggs, but was downregulated by ~3.9-fold in J2 Juveniles. The miR-279 in *M. incognita* has an orthologue in the infectious filarial nematode *Brugia malayi* [33], although its function in this parasitic nematode is not clear. Downregulation of miR-279 may result in reduced immune response. Eggs, external to the host, may be subject to an adverse chemical environment; however, the J2 juveniles, having penetrated the host, may shut down immune responses that they no longer need to deploy. The annotation of the *M. incognita* genome also suggested reduced immune effectors in *M. incognita* compared to the free-living *C. elegans* [5]. Similarly, miR-124, abundantly expressed in both eggs and J2s, also regulates the immune response. miR-124 is expressed in many sensory neurons and is highly conserved in both free-living and parasitic nematodes and in vertebrates [34]. miRNA 124 regulates many gene targets in the sensory nervous system and thus is critical for sensing environmental signals during infection of plants [15]. 

Another miRNA involved in immune response to pathogen infection and environmental toxicant stress is miR-252, the only significantly upregulated, conserved miRNA found in this study. miR-252 was upregulated ~4.6-fold in the J2 stage compared to the egg stage, with a TPM of 1541 in eggs and 7126 in J2 juveniles. The miR-252 loss-of-function mutant in *C. elegans* is more resistant to the infection from the pathogenic yeast *C. albicans*. miRNA may function downstream of the p38 mitogen-activated protein kinase (MAPK) or IGF-1/insulin-like pathways and regulate the innate immune response [35]. Therefore, upregulation of miR-252 may result in a reduced innate immune response, which is in consistent with the effects of miR-279 upregulation described above. It is also reported that the enhanced miR-252 expression is related to the increased cadmium tolerance in the water flea *Daphnia pulex* after multigenerational Cd exposure [36]. In addition, the miR-252 expression was increased by more than three-fold following dengue virus serotype 2 (DENV-2) infection in the Asian tiger mosquito *Aedes albopictus* [37]. All these findings suggest that miR-252 may play an important role in stress response during plant infection and defense. 

We performed a global gene target identification analysis and identified a total of 547 miRNA targets. Among these, 344 protein-coding genes were annotated using different databases. Based on GO analysis, miRNA targets protein-coding genes that are involved in various biological processes including metabolism, reproduction and development, signaling, and response to stimulus. Based on KEGG analysis, expressed miRNAs in *M. incognita* target pathways involved in genetic information processing, environmental information processing, cellular processes, and metabolism. The functions of the many miRNAs that were differentially expressed remain to be investigated in future studies, especially regarding their roles in development and parasitism.

## 4. Materials and Methods

### 4.1. M. incognita Culture and Sample Collection

The *M. incognita* eggs were obtained from Auburn University. After one week of cotton (*Gossypium hirsutum* L., cv “Texas Marker 1(TM-1)) seed germination, cotton seedlings were infected by *M. incognita* following our previously reported procedure [15,38]. Traditionally agricultural practice, including daily watering, was performed on cotton culture with the temperature of 30 ± 2 °C at daytime and 24 ± 2 °C at nighttime. After two months of culture (about two *M. incognita* life cycles), the RKN eggs were harvested from the infected cotton seedlings. Then, eggs were allowed to develop into J2 juveniles under aerobic conditions at room temperature. Both eggs and J2 larval were collected and immediately frozen at −80 °C until RNA extraction. Each developmental stage of samples was harvested for five biological replicates. Cotton seedling culture and RKN infection were performed in the greenhouse with regular agronomic practices, including watering. The egg hatching was performed in the growth chamber. 

### 4.2. RNA Extraction and Deep Sequencing

Total RNA was harvested from each sample using the mirVana™ miRNA Isolation Kit (Ambion Inc, Austin, TX, USA) according to the manufacturer’s instruction and our previous report with minor modification [15]. The quality and concentrations of each RNA samples were measured using a NanoDrop ND-1000 (Nanodrop Technologies, Wilmington, DE, USA).

The RNA samples were sent to Biomarker (Beijing, China) for small RNA sequencing. The sequencing protocol and bioinformatic analysis was similar to that of our previous report [39]. Summarizing briefly, all raw sequences from small RNAs were cleaned, including filtering out 5′ and 3′ adaptors and low-quality reads. Then, the raw sequences were categorized and read counts were calculated for each unique sequence. 

### 4.3. miRNA Identification and Expression

First, clean reads were matched to other small noncoding RNAs, including repeated RNAs, ribosomal RNAs (rRNAs), small nuclear RNAs (snRNAs), small nucleolar RNAs (snoRNAs), and transfer RNA (tRNA). These sequences were removed from the sequenced reads by identifying them in the Sanger RNA family database (Rfam 10.1, ftp://ftp.sanger.ac.uk/pub/databases/Rfam) [40] using LASTn-short alignment. The remaining sequences were further aligned against miRBase using our designed computational software, miRDeepFinder, to identify conserved miRNAs [39]. All small RNAs, except the other noncoding RNAs (ncRNAs), were compared against the *M. incognita* genome sequence to identify potential miRNA precursor sequences (pre-miRNA). The miRNAs were named according to publically accepted criteria. The expression level of each miRNA was also represented as transcripts read per mapped million sequenced reads (TPM).

The targets of both conserved and novel miRNAs were predicted using miRanda and RNAhybrid. The identified miRNA targets were further analyzed compare with NR, Swiss-Prot, GO, COG, KEGG, KOG, and Pfam databases. The GO and KEGG pathways were analyzed.

## Figures and Tables

**Table 1:** Summary of the raw and clean reads generated from deep sequencing from two developmental stages of the root-knot nematode (RKN).

Samples	Raw Reads	Low Quality	Containing ’N’ Reads	Length <18	Length >30	Clean Reads	Q30 (%)
Eggs	19,952,271	0	46	604,254	910,019	18,437,952	98.12
J2	17,526,380	0	171	1,611,967	524,776	15,389,466	98.13

Low quality reads: Bases with Quality value <30 larger than 20%; Containing ‘N’ reads: reads with unknown bases (N) more than 10%; Length <18: reads lower than 18 nt after adaptor removal; Length >30: reads more than 30 nt after adaptor removal; Clean reads: the number of reads with quality value ≥30; Q30(%): percentage of reads with quality value ≥30.

**Table 2:** Categorization of small RNAs sequenced in eggs and J2 larvae *.

Types	Eggs		J2	
	Number	Percentage (%)	Number	Percentage (%)
rRNA	3,840,307	20.83	2,573,694	16.72
scRNA	0	0.00	0	0.00
snRNA	0	0.00	0	0.00
snoRNA	3415	0.02	1359	0.01
tRNA	102,301	0.55	109,083	0.71
Repbase	2744	0.01	2417	0.02
Unannotated	14,489,185	78.59	12,702,913	82.54
Total	18,437,952	100.00	15,389,466	100.00

* The categorization of small RNAs were performed using the Bowtie software, which aligns clean reads to Silva, GtRNAdb, Rfam, and Repbase databases.

**Table 3:** The expression levels of 45 identified miRNAs in *M. incognita* eggs and J2 juveniles following cotton infection *.

miRNA	Eggs	J2 larvae	miRNA	Eggs	J2 larvae
miR22	8.71	2.88	miRN1	9.29	0.41
Let7	101.6	140.88	miRN10	2.9	2.06
miR1	244,810.84	426,558.94	miRN11	8.71	4.53
miR10227	320.48	109.16	miRN12	2.9	1.65
miR124	70,559.95	88,381.37	miRN13	43.54	25.54
miR239b	70,511.18	736.93	miRN14	42.38	27.19
miR2-3p	85,466.17	26,269.27	miRN15	2.9	1.24
miR252	1541.42	7125.89	miRN16	8.71	4.94
miR279	188,858.69	48,799.92	miRN17	4.06	2.06
miR3004	2.32	0.82	miRN18	10.45	5.77
miR4000	4.06	4.94	miRN19	1.16	137.17
miR4174	4.06	1.24	miRN2	13.35	2.47
miR4182	22.06	3.3	miRN20	216.55	77.44
miR429	23.22	11.95	miRN21	14.51	2.06
miR4738	6.97	0	miRN3	4.06	0
miR57-5p	4555.18	2055.09	miRN4	8.71	2.47
miR7029	80.12	51.08	miRN5	2.9	0
miR7904	21,868.45	10,275.88	miRN6	2.32	1.24
miR7954	30.19	8.24	miRN7	33.09	21.83
miR8411	29.03	0.82	miRN8	39.48	24.3
miR87	86,106.54	70,471.29	miRN9	199.14	140.05
miR8917	29.03	15.65			
miR92	221,662.23	317,693.78			
miR993-3p	2726.37	796.25			

* The expression levels were presented as TPM (the total transcripts read number of per one million total sequenced read that were mapped to the *M. incognita* genome sequence).

**Table 4:** Differentially expressed miRNAs between RKN eggs and J2 larvae.

miRNA	S01-Eggs	S02-J2	*p*-Value	Log2FC	Regulated
miR7954	30.2	8.2	<0.0001	−1.874	down
miR57-5p	4555.2	2055.1	<0.0001	−1.148	down
miR7904	21,868.4	10,275.9	<0.0001	−1.09	down
miR4182	22.1	3.3	<0.0001	−2.743	down
miR2-3p	85,466.2	26,269.3	<0.0001	−1.702	down
miR279	188,858.7	48,799.9	<0.0001	−1.952	down
miR8411	29	0.8	<0.0001	−5.139	down
miRN19	1.2	137.2	<0.0001	6.8843	up
miR239b	70,511.2	736.9	<0.0001	−6.58	down
miR993-3p	2726.4	796.3	<0.0001	−1.776	down
miR10227	320.5	109.2	<0.0001	−1.554	down
miR252	1541.4	7125.9	<0.0001	2.2088	up
miRN20	216.6	77.4	<0.0001	−1.484	down
miRN21	14.5	2.1	0.000001	−2.817	down
miRN1	9.3	0.4	0.000003	−4.495	down
miR4738	7	0	0.00001	−26.05	down
miRN2	13.4	2.5	0.00001	−2.434	down
miRN3	4.1	0	0.0009	−25.28	down
miRN4	8.7	2.5	0.0017	−1.817	down
miR22	8.7	2.9	0.0031	−1.595	down
miRN5	2.9	0	0.0051	−24.79	down

**Table 5:** Annotation of miRNA targets against different databases.

Database	Annotated Number	300 ≤ Length < 1000 *	Length ≥1000 *
COG	141	46	30
GO	180	64	60
KEGG	137	47	45
KOG	203	74	69
Pfam	260	95	81
Swissprot	184	66	68
eggNOG	268	84	90
nr	286	99	88
All	344	117	92

* The length means the defined gene target size.
